# Seasonal and Regional Differences in Gene Expression in the Brain of a Hibernating Mammal

**DOI:** 10.1371/journal.pone.0058427

**Published:** 2013-03-20

**Authors:** Christine Schwartz, Marshall Hampton, Matthew T. Andrews

**Affiliations:** 1 Department of Biology, University of Minnesota Duluth, Duluth, Minnesota, United States of America; 2 Department of Mathematics and Statistics, University of Minnesota Duluth, Duluth, Minnesota, United States of America; Wayne State University, United States of America

## Abstract

Mammalian hibernation presents a unique opportunity to study naturally occurring neuroprotection. Hibernating ground squirrels undergo rapid and extreme physiological changes in body temperature, oxygen consumption, and heart rate without suffering neurological damage from ischemia and reperfusion injury. Different brain regions show markedly different activity during the torpor/arousal cycle: the cerebral cortex shows activity only during the periodic returns to normothermia, while the hypothalamus is active over the entire temperature range. Therefore, region-specific neuroprotective strategies must exist to permit this compartmentalized spectrum of activity. In this study, we use the Illumina HiSeq platform to compare the transcriptomes of these two brain regions at four collection points across the hibernation season: April Active, October Active, Torpor, and IBA. In the cerebral cortex, 1,085 genes were found to be differentially expressed across collection points, while 1,063 genes were differentially expressed in the hypothalamus. Comparison of these transcripts indicates that the cerebral cortex and hypothalamus implement very different strategies during hibernation, showing less than 20% of these differentially expressed genes in common. The cerebral cortex transcriptome shows evidence of remodeling and plasticity during hibernation, including transcripts for the presynaptic cytomatrix proteins bassoon and piccolo, and extracellular matrix components, including laminins and collagens. Conversely, the hypothalamic transcriptome displays upregulation of transcripts involved in damage response signaling and protein turnover during hibernation, including the DNA damage repair gene *RAD50* and ubiquitin E3 ligases *UBR1* and *UBR5*. Additionally, the hypothalamus transcriptome also provides evidence of potential mechanisms underlying the hibernation phenotype, including feeding and satiety signaling, seasonal timing mechanisms, and fuel utilization. This study provides insight into potential neuroprotective strategies and hibernation control mechanisms, and also specifically shows that the hibernator brain exhibits both seasonal and regional differences in mRNA expression.

## Introduction

**Figure 1:**
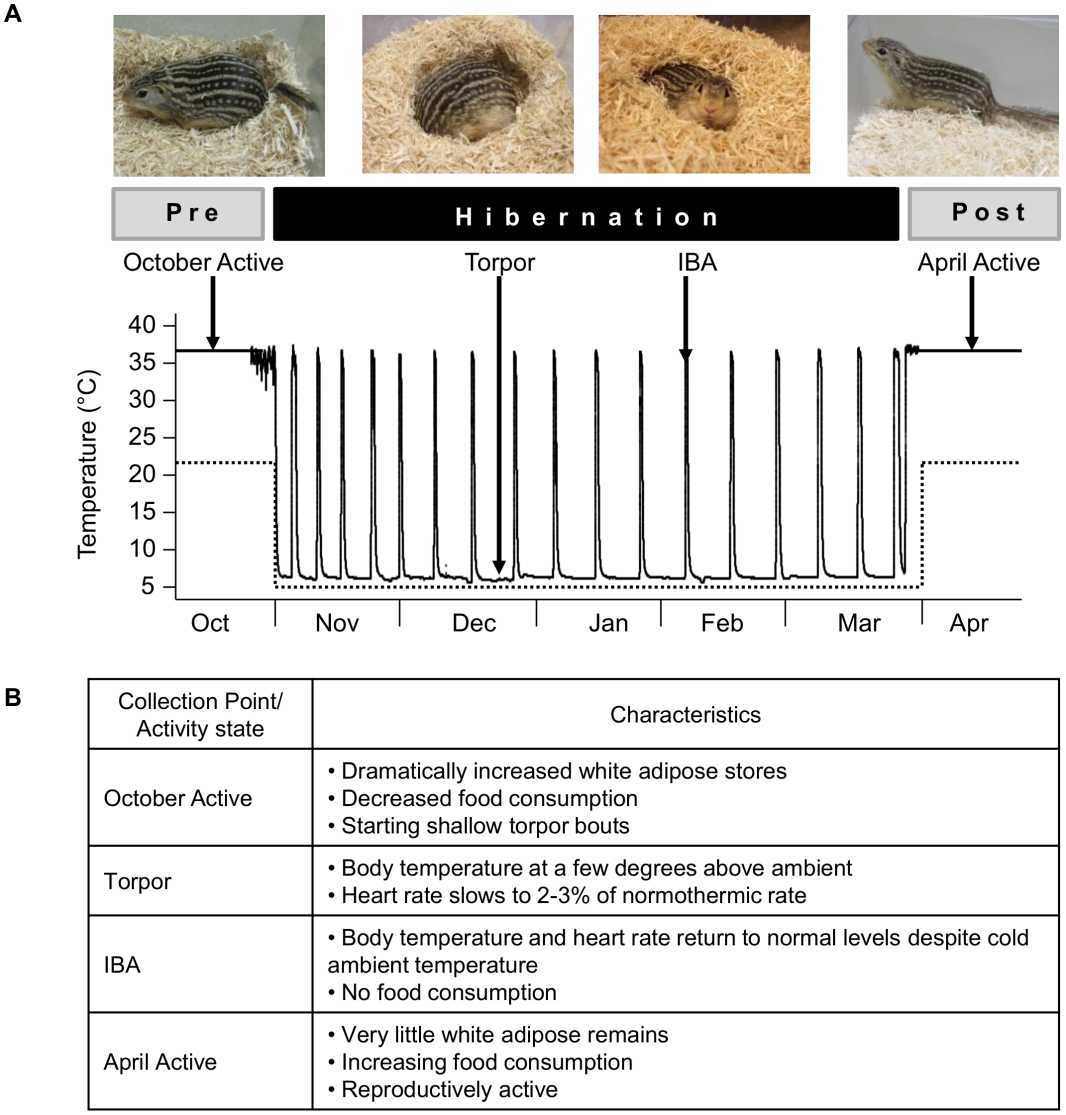
Animal appearance and physiological characteristics at experimental collection points. *A*. Representative photographs of thirteen-lined ground squirrels at each of the four collection points used in this study: pre-hibernation (October Active), Torpor, IBA, and post-hibernation (April Active). The bottom graph displays a trace of core body temperature from a single animal measured by a surgically implanted transmitter (solid black line), along with the controlled ambient temperature of the same ground squirrel’s environmental chamber (dashed line) over the course of a hibernation season. Arrows indicate representative body temperatures at each of the four collection points. *B*. A brief description of the behavioral and physiological characteristics of the ground squirrels at each point. IBA: Interbout arousal.

Hibernation is an adaptive phenotype which allows specific mammals to survive long periods at near-freezing temperatures with little or no food. During hibernation, mammals enter a period of heterothermy, characterized by lengthy periods of low body temperature, metabolism, and heart rate called torpor bouts, interspersed with short periodic interbout arousals (IBAs) to normothermia (Figure 1). During torpor, thirteen-lined ground squirrels (*Ictidomys tridecemlineatus*) show a heart rate less than 5% of their normothermic rate, use only 2–3% of their normal oxygen levels, and maintain a body temperature of 2–10°C, physiological characteristics that would result in death for most mammals [1]. Consequently, the brain is exposed to extreme fluctuations in temperature and blood flow during hibernation. Specifically, cerebral blood flow in thirteen-lined ground squirrels is reduced by 90% during torpor, levels that are normally ischemic in non-hibernators [2]. However, no histological abnormalities are seen in any brain region after arousal from torpor in this ground squirrel species [2]. Identifying the molecular mechanisms orchestrating the extreme changes in physiology during hibernation, and the accompanying natural neuroprotection, could be an important contribution to the development of therapeutic strategies for the treatment and prevention of brain injury.

In addition to the striking physiological fluctuations affecting the whole brain during hibernation, there are also regional differences in activity during torpor and IBA. Analysis of electroencephalography, immediate early gene expression, and 2-deoxyglucose uptake indicate that the cerebral cortex is the first area of the brain to lose activity as the animal goes into torpor and the last area to regain activity upon arousal to IBA, while parts of the hypothalamus remain active throughout the hibernation season, even in torpor [3], [4], [5]. The cerebral cortex undergoes extensive synaptic structural changes during torpor [6], [7], which could be contributing to, or a result of, the cessation of activity in this brain region. However, this synaptic plasticity has not been examined in the continuously active hypothalamus. The hypothalamus functions to maintain homeostasis, and is involved in many processes potentially important for hibernation and preparation for hibernation, including food intake/satiety signaling [8], [9], [10], circadian rhythms [11], sleep [12], body temperature [13], and hormone production [14], [15]. Thus, the hypothalamus could play a major role in both the seasonal initiation of hibernation and the orchestration and maintenance of torpor/IBA cycles. The region-specific regulation of activity in the brain during hibernation suggests that there could be substantial differences in the molecular mechanisms underlying the control of hibernation in different areas of the brain. To our knowledge, no one has examined hibernation-specific changes in overall gene expression across different brain regions.

Here, we used next generation sequencing (Illumina HiSeq 2000) to sequence the transcriptome of the hypothalamus and cerebral cortex at four collection points: pre-hibernation (October Active), Torpor, IBA, and post-hibernation (April Active) (Figure 1). The transcriptome provides a comprehensive summary of all the genes expressed at each collection point. The widespread appearance of the hibernator phenotype in Class Mammalia suggests that hibernators do not have a specific set of genes dedicated to hibernation, but rather they use differential expression of genes that exist in most mammals [16]. Therefore, the hibernator brain provides an excellent and unique opportunity to uncover genes both relevant and potentially targeted to human health, particularly in relation to neuroprotective therapies. Because these two areas of the brain are so distinctly different both in function and activity during hibernation, we predict that comparing mRNA levels between both regions and across collection points will reveal genes involved in region-specific survival strategies, along with providing insight into how hibernation is seasonally controlled and regulated on the transcriptional level in the brain.

## Results

### Overview

This study examined the transcriptome of the ground squirrel cerebral cortex and hypothalamus over the course of the hibernation season. Each brain region was first independently characterized by examining the most abundant and most region-specific transcripts in each region. The brain regions were then examined over the course of the hibernation season to determine how mRNA composition changed. Table 1 displays the overall mRNA numbers resulting from the transcriptomic analysis, showing that, in general, the two brain regions are over 90% identical in terms of mRNA diversity. However, the number of differentially expressed genes across collection points that were shared between the two brain regions was less than 20% of the total.

**Table 1:** Overview of Illumina results.

Tissue	Protein-coding genes identified	Differentially expressed genes
Cerebral cortex	8,981	1,085
Hypothalamus	9,283	1,063
Shared between both tissues	8,528	204

All genes identified have more than 100 average counts in at least one collection point. Identified genes are considered differentially expressed if they have at least a 50% change between the highest and lowest levels at different collection points and have an FDR<0.05.

### Regional Specificity of Gene Expression

**Figure 2:**
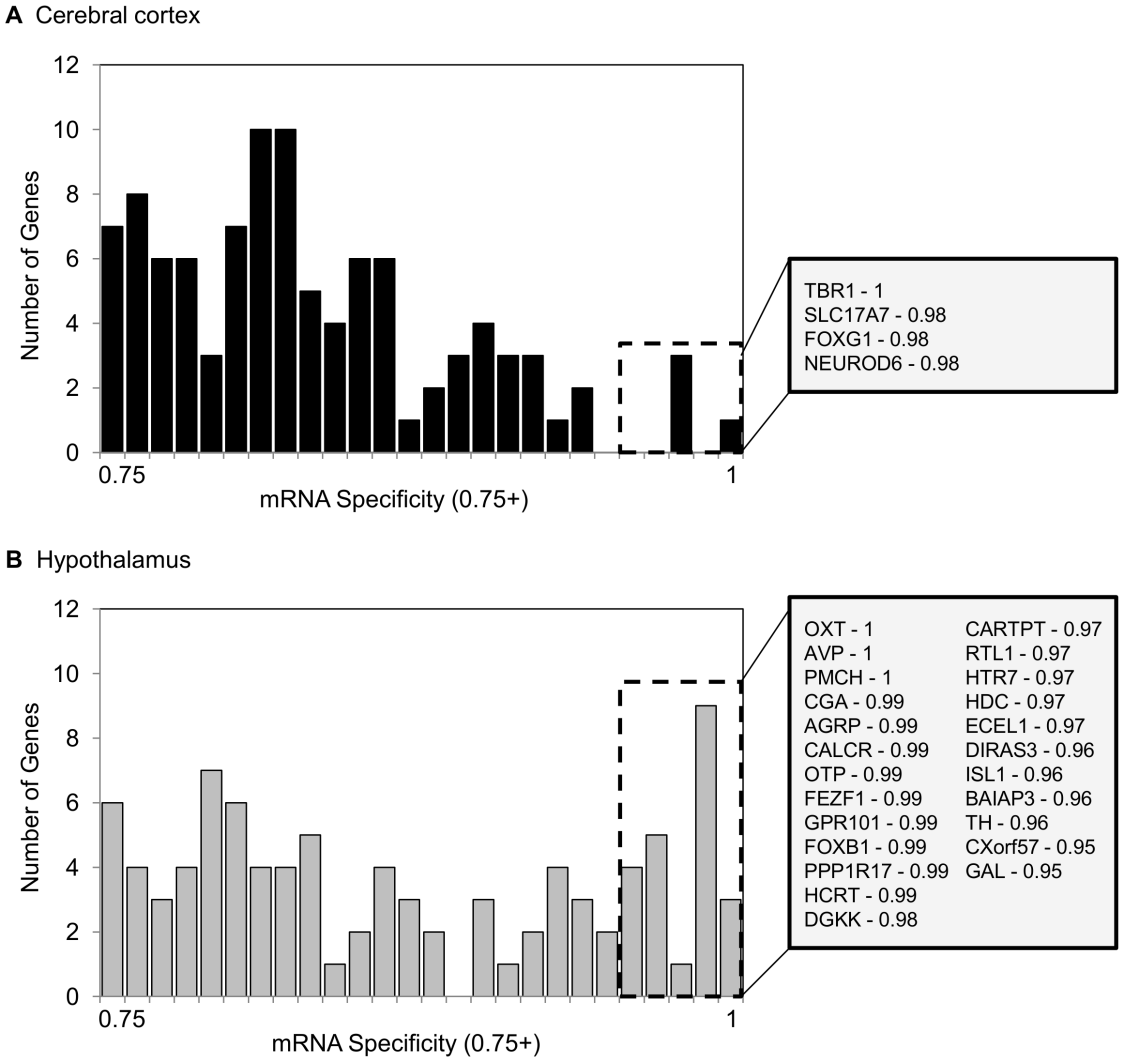
Regional gene expression specificity. Specificity values ranging from 0.75–1.0 are mapped in histograms for (*A*) cerebral cortex and (*B*) hypothalamus. An expression specificity value of 1.0 means that 100% of the reads for that mRNA were found in the indicated region. mRNAs with an expression specificity value of 0.95 or higher are denoted by a dashed box and listed in the gray box to the right of each histogram. The corresponding specificity values are listed after each mRNA. Gene names listed are HGNC designations.

The hypothalamus exhibited higher mRNA specificity than the cerebral cortex, with six times as many mRNAs that were at least 95% specific to this region (Figure 2). This includes the precursors for the pituitary hormones oxytocin and vasopressin (*OXT* and *AVP*), and pro-melanin-concentrating hormone (*PMCH*), the precursor for melanin-concentrating hormone, which were not found in the cerebral cortex. T-box brain protein 1 (*TBR1*) was the only mRNA that was 100% specific for the cerebral cortex.

### Most Abundant Transcripts

**Figure 3:**
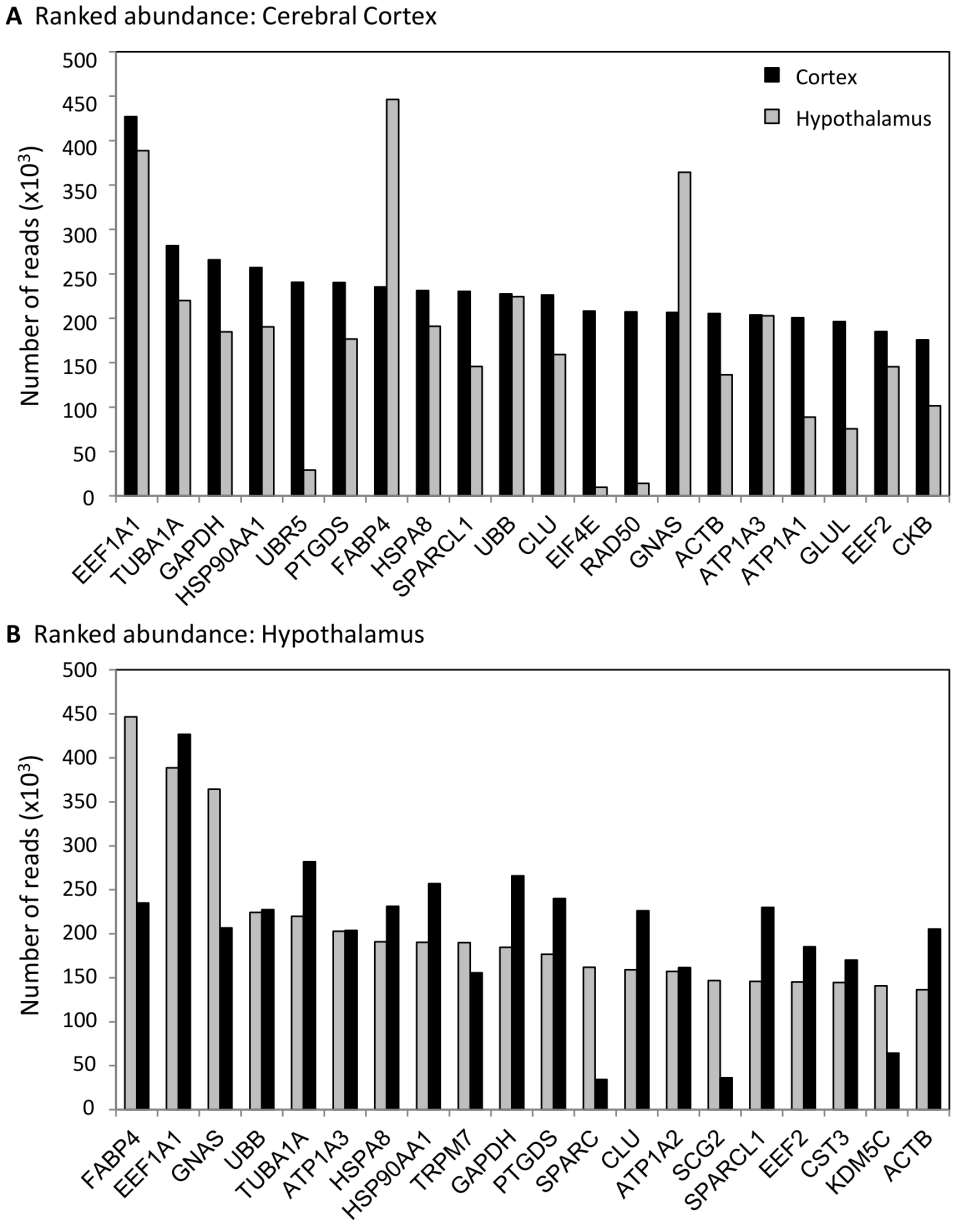
Most abundant mRNAs. Abundances for the top 20 mRNAs in (*A*) cerebral cortex and (*B*) hypothalamus are displayed, along with the accompanying abundance from the other brain region for comparison. Cerebral cortex mRNA abundances are shown in black, hypothalamus abundances are shown in gray. Gene names listed are HGNC designations.

Figure 3 displays and ranks the 20 most abundant transcripts in the (A) cerebral cortex and (B) hypothalamus, 14 of which are shared between the two brain regions. Ubiquitin protein ligase E3 component n-recognin 5 (*UBR5*), eukaryotic translation initiation factor 4E (*EIF4E*), RAD50 (*S. cerevisiae*) homolog (*RAD50*), Na^+^/K^+^ transporting ATPase, alpha 1 polypeptide (*ATP1A1*), glutamate-ammonia ligase (*GLUL*), and brain creatine kinase (*CKB*) are more abundant in cerebral cortex. Transient receptor potential cation channel, subfamily M, member 7 (*TRPM7*), secreted protein, acidic, cysteine-rich (*SPARC*), Na^+^/K^+^ transporting ATPase, secretogranin II (*SCG2*), and lysine (K)-specific demethylase 5C (*KDM5C*) are more abundant in the hypothalamus. Alpha 2 polypeptide (*ATP1A2*) and cystatin C (*CST3*) are within the top 20 most abundant transcripts in the hypothalamus and not in cerebral cortex, but they are actually more abundant in the cerebral cortex.

Several of the most abundant transcripts are also differentially expressed (FDR<0.05) over the four collection points (Table S1). *UBR5* and *RAD50* are differentially expressed in both brain regions. *TRPM7* and prostaglandin D2 synthase (*PTGDS)* are both differentially expressed in cerebral cortex. Fatty acid binding protein 4 (*FABP4*), *EIF4E*, *CKB*, and *SPARC* are all differentially expressed in hypothalamus.

### Differential Expression

**Figure 4:**
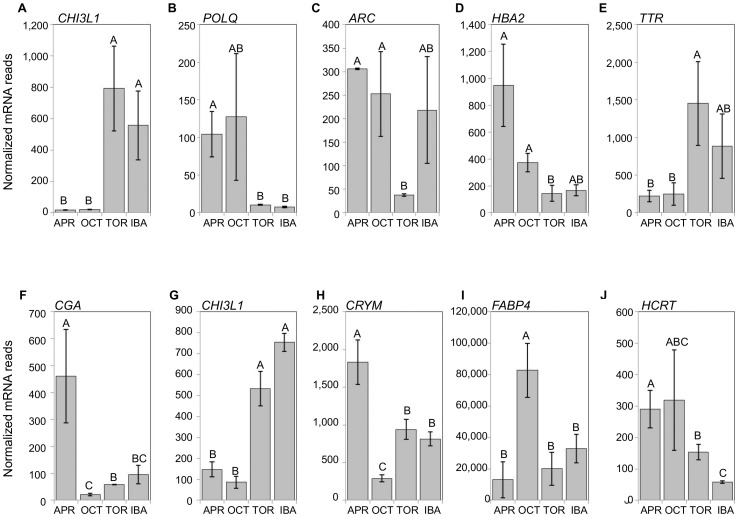
Extreme changes in gene expression across collection points. Differentially expressed transcripts displaying the largest fold changes in cerebral cortex (A-E) and hypothalamus (F-J). Error bars represent standard error of the mean. All mRNAs had an FDR<0.05. The letters above each bar represent post hoc pair-wise comparisons to determine significance between collection points. Any collection point not connected by the same letter is significantly different (FDR<0.05). In cerebral cortex, *CHI3L1* (A) exhibited the highest fold change, followed by *POLQ* (B), *ARC* (C), *HBA2* (D), and *TTR* (E). In hypothalamus, *CGA* (F) exhibited the highest fold change, followed by *CHI3L1* (G), *CRYM* (H), *FABP4* (I), and *HCRT* (J). Gene names listed are HGNC designations.

Only 20% of differentially expressed genes (FDR<0.05) are shared between the two brain regions (Table 1). The expression patterns across collection points of the top five mRNAs with the highest fold changes in each brain region are shown in Figure 4. Only one transcript, chitinase 3-like 1 (*CHI3L1*), is highly differentially expressed (fold change >4) in both brain regions. This transcript exhibits the highest fold change in the cerebral cortex (40.3), and the second highest fold change (8.6) in the hypothalamus. Both brain regions have high *CHI3L1* counts during the hibernation collection points and very low counts in the pre- and post-hibernation collection points, indicating that this transcript is very hibernation-specific in the brain. The most highly differentially expressed gene in the hypothalamus is glycoprotein hormones, alpha polypeptide (*CGA*), with a 20.7 fold change.

### Functional Analysis – Region-specific Hibernation Strategies

**Figure 5:**
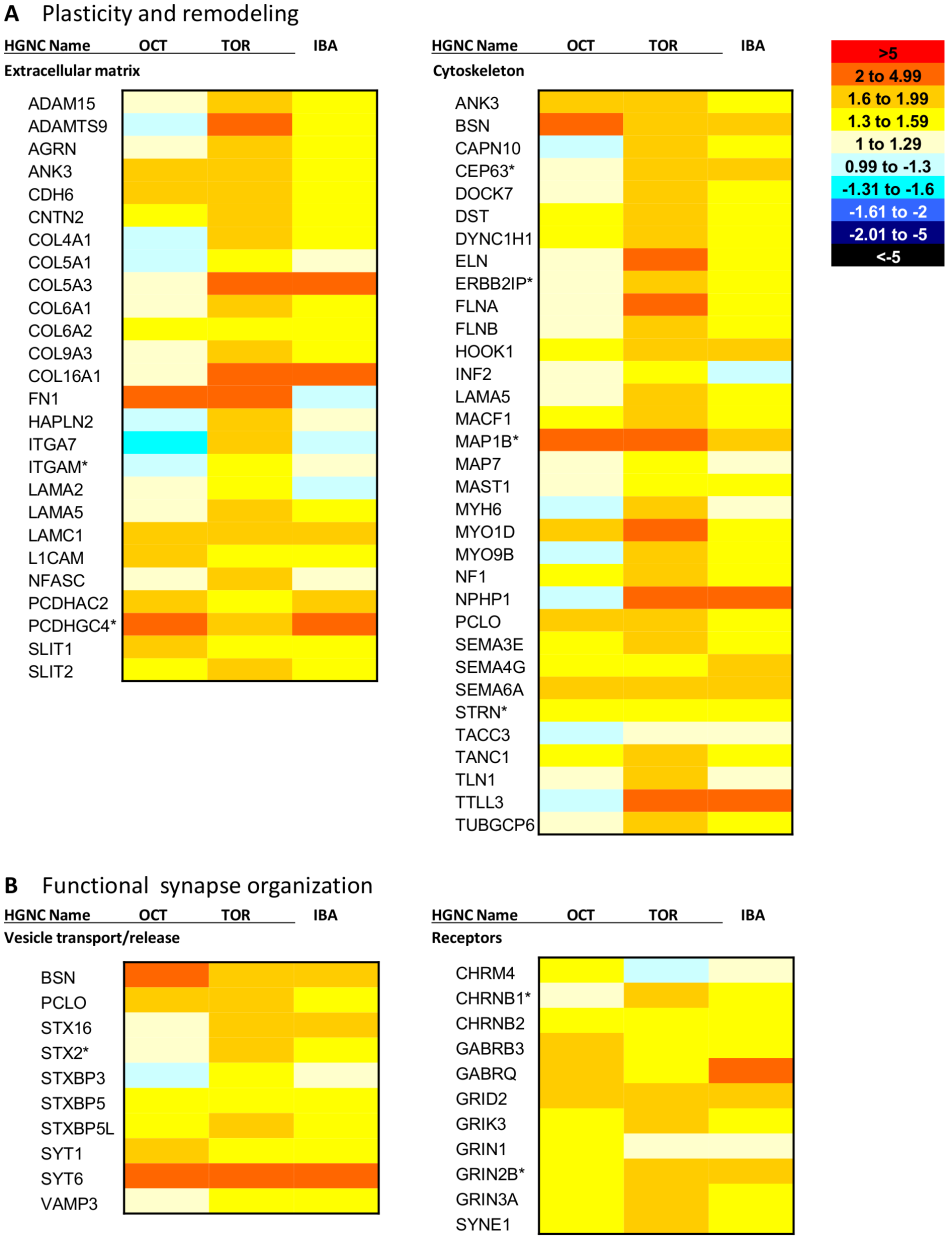
Evidence of dynamic structural changes during hibernation in cerebral cortex. Heatmap showing hibernation-specific elevation of mRNAs involved in (*A*) plasticity and remodeling and (*B*) synaptic transmission in the cerebral cortex. *A* is divided into two broad categories: extracellular matrix and cytoskeleton. *B* is also divided into two categories: vesicle transport/release and receptors. October (OCT), Torpor (TOR) and IBA collection points are all shown relative to the April collection point (expression = 1). Each mRNA shown is differentially expressed in the cerebral cortex (FDR<0.05). The heatmap scale is provided on the upper right of *A*. Red colors indicate higher expression than April, while blue colors indicate lower expression than April. Transcripts labeled with an asterisk show the same differential expression pattern in hypothalamus as well. Gene names listed are HGNC designations. IBA: interbout arousal.

**Figure 6:**
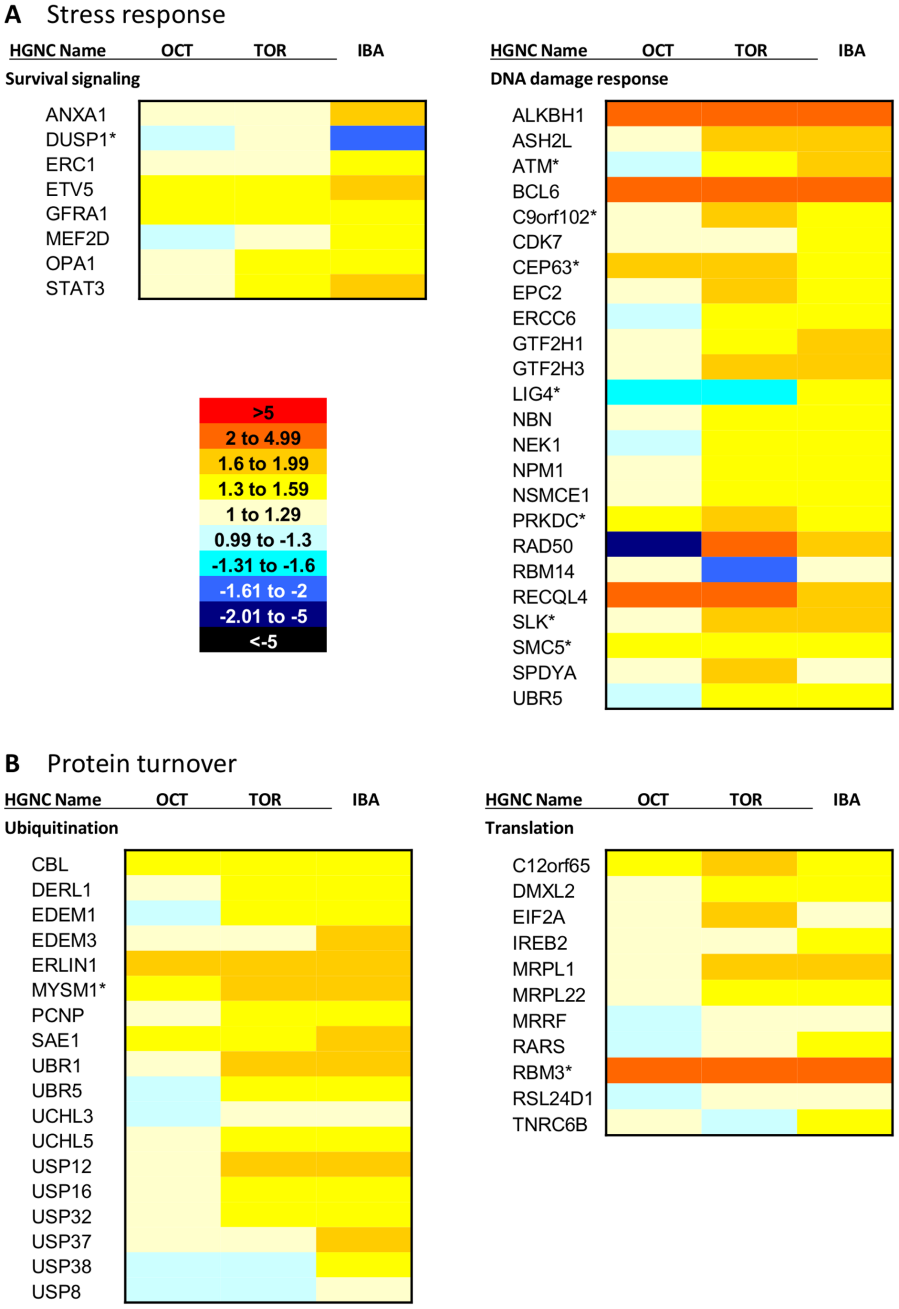
Damage repair and protein turnover during hibernation in hypothalamus. This heatmap shows hibernation-specific elevation of (*A*) stress response and (*B*) protein turnover mRNAs in the hypothalamus. *A* is divided into two broad categories: survival signaling and DNA damage response. *B* is also divided into two categories: ubiquitination and translation. October (OCT), Torpor (TOR) and IBA collection points are all shown relative to the April collection point (expression = 1). Each mRNA shown is differentially expressed in the hypothalamus (FDR<0.05). The heatmap scale is provided on the bottom left of *A*. Red colors indicate higher expression than April, while blue colors indicate lower expression than April. Transcripts labeled with an asterisk show the same differential expression pattern in cerebral cortex as well. Gene names listed are HGNC designations. IBA: interbout arousal.

Analysis of the differentially expressed transcripts using the Database for Annotation, Visualization and Integrated Discovery (DAVID) [17] and accompanying literature searches provided an overall picture of the overrepresented functions in each brain region during hibernation, and in some cases, pre-hibernation. In the cerebral cortex, there is evidence of plasticity and remodeling occurring, along with upregulation of transcripts important for organization of synaptic transmission (Figure 5), supporting previous findings that synaptic connectivity is reduced during torpor but returns during IBA [6], [7]. In the hypothalamus, there is evidence of stress response and protein turnover (Figure 6), consistent with reports of hypothalamic activity throughout hibernation [3], [4], [5].

Plasticity, remodeling, and organization of synaptic transmission in the pre-hibernation and hibernation collection points in the cerebral cortex appear to be accomplished in several ways (Figure 5). First, genes involved in cytoskeletal morphogenesis, including presynaptic cytomatrix protein transcripts Bassoon (*BSN*) and Piccolo (*PCLO*), and postsynaptic microtubule-associated protein 1B (*MAP1B*) are elevated during October, torpor, and IBA. Second, the cerebral cortex transcriptome reveals evidence of extracellular modulation. Transcripts of structural extracellular matrix (ECM) components show elevated levels during hibernation, including collagens (*COL9A3, 4A1, 6A1, 5A1*), laminins (*LAMA3, LAMA5*), and hyaluronan and proteoglycan link protein 2 (*HAPLN2*), which all show peak expression in torpor. Additionally, cell adhesion molecules, including integrins (*ITGA7*, *ITGAM*), contactin 2 (*CNTN2)*, and agrin (*AGRN*), also show highest expression during torpor. Finally, transcripts involved in synaptic transmission are elevated during hibernation, specifically including transcripts involved in vesicle transport and release, such as syntaxin 2 (*STX2*), and vesicle-associated membrane protein 3 (*VAMP3*), which are both elevated in torpor and IBA. In addition, transcripts for synaptic receptors, including NMDA glutamate receptors *GRIN2B* and *GRIN3A*, are elevated in October, torpor and IBA.

In contrast to the plasticity and remodeling occurring in the cerebral cortex during hibernation, the hypothalamus appears to upregulate a stress response including survival signaling and repair mechanisms, along with protein turnover (Figure 6). Expression of neuronal survival-related myocyte enhancer factor 2D (*MEF2D*) and signal transducer and activator of transcription 3 (*STAT3*) show highest expression levels during IBA. Transcript levels of DNA damage response controller ataxia telangiectasia mutated (*ATM*) and double-strand break repair gene *RAD50* are increased during torpor and IBA. In addition to repair mechanisms, the hypothalamus also shows evidence of ubiquitin-dependent protein catabolism. E3 ubiquitin ligases *UBR1* and *UBR5* are upregulated during torpor and IBA, along with deubiquinating *USP12*, *USP16*, and *USP32*. Genes promoting translation are increased as well, in particular eukaryotic translation initiation factor 2A (*EIF2A*), which peaks in torpor, and cold-inducible RNA binding motif protein 3 (*RBM3*), which is elevated in October, torpor, and IBA.

### Functional Analysis - Hibernation Induction and Maintenance

**Figure 7:**
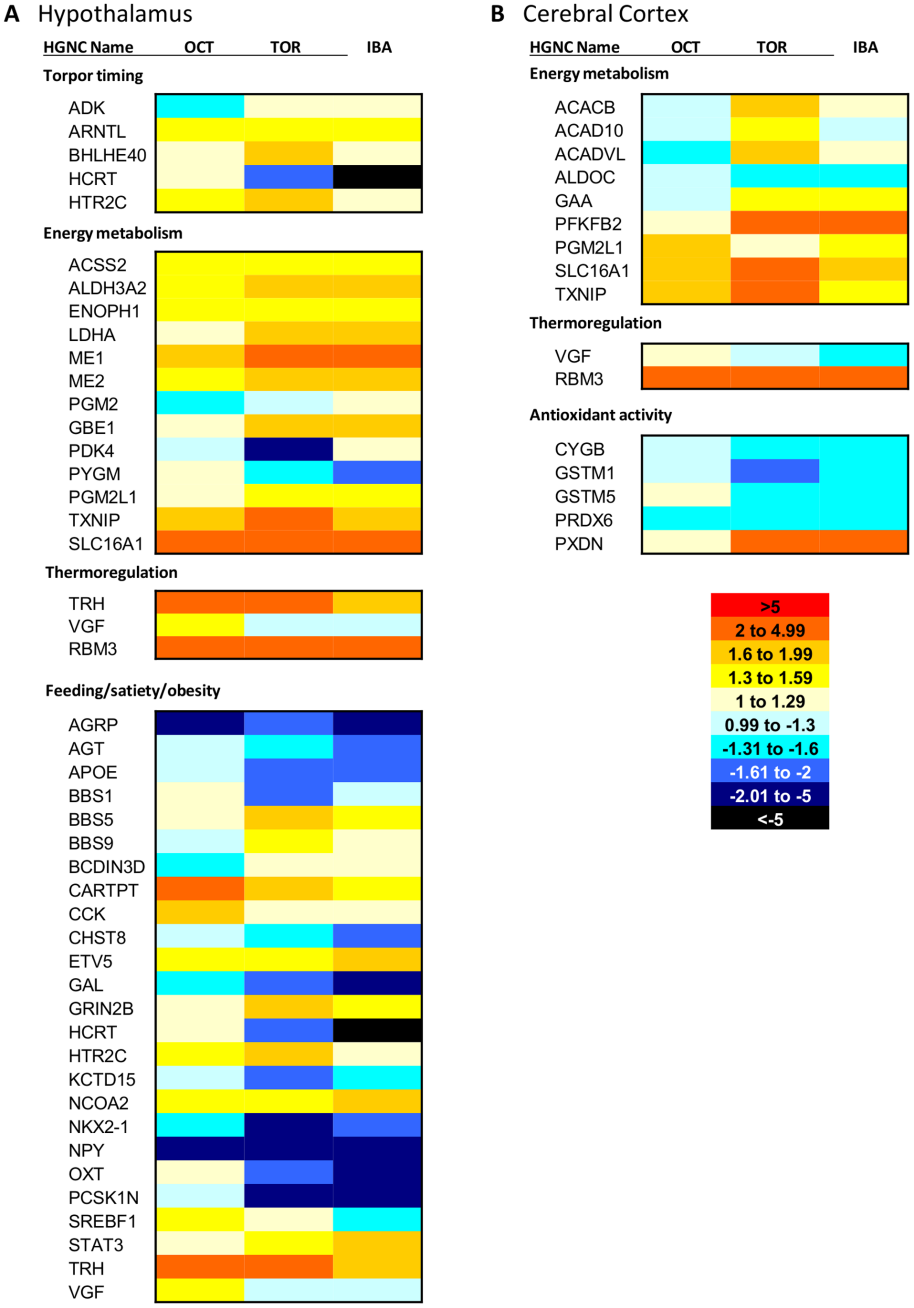
Candidate genes potentially contributing to hibernation phenotype. This heatmap shows hibernation candidate genes in (*A*) hypothalamus and (*B*) cerebral cortex. *A* is divided into four categories: torpor timing, energy metabolism, thermoregulation, and feeding/satiety/obesity. *B* is divided into three categories: energy metabolism, thermoregulation, and antioxidant activity. October (OCT), Torpor (TOR) and IBA collection points are all shown relative to the April collection point (expression = 1). Each mRNA shown is differentially expressed in the hypothalamus (FDR<0.05). The heatmap scale is provided on the lower right of *B*. Red colors indicate higher expression than April, while blue colors indicate lower expression than April. Gene names listed are HGNC designations. IBA: interbout arousal.

The hypothalamus controls many homeostatic processes important for hibernation, including feeding behavior and satiety signaling. Transcriptomic analysis revealed several genes involved in feeding behavior that are differentially expressed across collection points (Figure 7A). Orexigenic agouti related protein (*AGRP*) and neuropeptide Y (*NPY*) show low expression during the pre-hibernation period when food intake is declining, while anorexigenic cocaine and amphetamine regulated transcript prepropeptide (*CARTPT*) and thyrotropin-releasing hormone (*TRH*) show high expression levels. Conversely, in the post-hibernation period, where food intake is high, *AGRP* and *NPY* have their highest expression levels, while *CARTPT* and *TRH* are low. During hibernation (both torpor and IBA), *AGRP* and *NPY* remain low, while *CARTPT* and *TRH* are at moderate levels. Orexigenic hypocretin (*HCRT*) is elevated in both the pre- and post-hibernation collection points, but is low during hibernation.

Thermoregulation is also controlled by the hypothalamus and plays an important role in hibernation, as body temperatures cycle between near-freezing in torpor to normal levels in IBA. *TRH* and *VGF*, known to be involved in body temperature regulation, both peak in the pre-hibernation collection point in the hypothalamus (Figure 7A). Additionally, *RBM3*, which promotes translation at cold body temperatures [18], is elevated during pre-hibernation and hibernation (torpor and IBA) in both hypothalamus and cerebral cortex (Figure 7).

Circadian rhythms could also be playing a role in hibernation timing and are controlled by the hypothalamus. Two circadian genes, aryl hydrocarbon receptor nuclear translocator-like (*ARNTL*) and basic helix-loop-helix family, member e40 (*BHLHE40*), are differentially expressed in the hypothalamus (Figure 7A). *ARNTL* is highest during IBA but lowest during torpor. *BHLHE40* peaks during torpor and is lowest outside of hibernation.

Prior work indicates that antioxidant strategies used by natural hibernators could play an important role in protecting the brain from damage [19], [20], [21]. Only one differentially expressed transcript with reported antioxidant function, peroxidasin homolog (*Drosophila*) (*PDXN*), was upregulated in the cerebral cortex during torpor and IBA (Figure 7B). All others were highly expressed outside of hibernation.

Finally, there is evidence of a fuel switch from glucose to ketones during hibernation in the brain (Figure 7). The ketone transporter *SLC16A1* is upregulated in October, torpor, and IBA in both cerebral cortex and hypothalamus, supporting previous work [22]. Fatty acid beta-oxidation pathway genes acyl-CoA dehydrogenase family, member 10 (*ACAD10*), acyl-CoA dehydrogenase, very long chain (*ACADVL*), and acetyl-CoA carboxylase beta (*ACACB*) are elevated in the cerebral cortex during torpor, while acyl-CoA synthetase short-chain family member 2 (*ACSS2*) is elevated in the hypothalamus during October, torpor, and IBA. Additionally, glycolytic enzyme aldolase C, fructose-bisphosphate (*ALDOC*) is highest in April in the cerebral cortex, while 6-phosphofructo-2-kinase/fructose-2,6-biphosphatase 2 (*PFKFB2*), which can negatively regulate glycolysis, is upregulated during torpor and IBA. However, there is also some evidence of glucose utilization in hibernation, with glycogen degrading acid alpha glucosidase (*GAA*) elevated in hypothalamus and anaerobic glycolysis component lactate dehydrogenase A (*LDHA*) elevated in cerebral cortex during both torpor and IBA.

## Discussion

### Overview

The cerebral cortex and hypothalamus show strikingly different transcriptional profiles across the hibernation season, particularly in terms of neuroprotection and fuel utilization, supporting their different functions and activity patterns. The cerebral cortex exhibits remodeling and plasticity during hibernation, along with evidence of synapse functional organization, which is not seen in the hypothalamus. The hypothalamus shows a defense strategy consisting of DNA repair, ubiquitin-mediated protein turnover, and survival signaling. Evidence of a hibernation fuel switch from glucose to ketones is evident in both brain regions, but glucose also appears to be utilized, particularly in the hypothalamus. The hypothalamic transcriptome provides some potential clues about the molecular signaling underlying hibernation physiology, particularly feeding behavior/satiety, thermoregulation, and timing of torpor bouts. Additionally, in contrast to the striking differences in differentially expressed genes between the cerebral cortex and hypothalamus, comparison of the overall most abundant mRNAs reveals that the two brain regions are very similar. However, in terms of gene expression specificity, the hypothalamus exhibits many more unique transcripts than the cerebral cortex.

### Plasticity and Remodeling in Hibernation – Example of a Cerebral Cortex-specific Phenotype

Analysis of the cerebral cortex transcriptome revealed evidence of remodeling and plasticity during hibernation. Previous accounts reported a loss of synaptic connectivity during torpor in this region, which is regained during IBA, specifically through changes in dendrite structure [6] and synaptic protein dynamics [7]. This reconnection must occur in less than three hours to be completed by the onset of normothermia [23]. A comparison of pre- and postsynaptic protein expression in the hibernator brain revealed that proteins on both sides of the synapse dissociate during torpor, but reconnect during IBA, with no overall loss of protein levels [7]. However, examination of the breakdown products of one synaptic protein in the synaptosomal fraction of torpid ground squirrel brains revealed that proteolysis is occurring [7]. Because there is no net loss of total protein, there must be protein synthesis to replace the lost proteins, underscoring the importance of gene expression during this time. We propose that synaptic plasticity during hibernation is a method of neuroprotection, preventing damage to the neurons of the cerebral cortex as the animal cycles through physiological extremes during hibernation. The cerebral cortex transcriptome reveals gene products potentially involved in this synaptic plasticity (Figure 5).

#### Cytoskeletal morphogenesis

Physical separation of the pre- and postsynaptic components of neurons could provide neuroprotection by removing the potential for signal transmission and thus eliminating the possibility of excitotoxicity upon arousal from torpor. Evidence for cytoskeleton morphogenesis is present in the cerebral cortex transcriptome, including synapse-specific transcriptional changes (Figure 5A). The transcripts for the presynaptic cytomatrix proteins Piccolo (*PCLO*) and Bassoon (*BSN*) are elevated during the hibernation (torpor and IBA) and pre-hibernation periods. These proteins organize the active zones of presynaptic neurons, where synaptic vesicle machinery is organized and the vesicles are released [24]. Normally, the presynaptic active zone clusters with the postsynaptic density of the receiving neuron, forming a functional synapse. Piccolo has been shown to dissociate from postsynaptic density proteins during torpor, but returns during IBA [7]. This suggests that the entire active zone is unorganized and/or retracted during torpor and reorganized upon arousal to IBA, eliminating the neuron’s ability to release synaptic vesicles and effectively propagate a signal. There is also upregulation of transcripts involved in vesicle transport and release during hibernation, including synaptotagmin 1, which is important for vesicle fusion and exocytosis [25]. This could be important to reestablish a functional synapse during IBA.

Additionally, two transcripts associated with postsynaptic cytoskeleton morphology are also elevated during hibernation (Figure 5A). *MAP1B* promotes dendrite outgrowth and maturation [26]. Tetratricopeptide repeat, ankyrin repeat and coiled-coil containing 1 (*TANC1*) has also been shown to regulate dendritic spines and excitatory synapses [27]. Both *MAP1B* and *TANC1* are elevated in the pre-hibernation and hibernation collection points compared to April. These transcripts could be orchestrating a similar dissociation and reconnection of the postsynaptic density during hibernation, and thus preventing signal reception, particularly through modification of available receptors. Synaptic plasticity through receptor modulation is a key component of the neuronal plasticity mechanisms long term potentiation (LTP), where signal transmission is enhanced, and long term depression (LTD), where the efficacy of transmission is reduced. There is evidence of a similar modification of receptor availability in hibernation. Spectrin repeat containing, nuclear envelope 1 (*SYNE1*) is highest during torpor (Figure 5B) and is involved in endocytosis of membrane proteins, including NMDA and AMPA glutamate receptors [28]. Additionally, *ARC* (activity-regulated cytoskeleton-associated protein) is an immediate early gene associated with synaptic plasticity that localizes to dendrites and functions in AMPA receptor endocytosis [29]. *ARC* transcript levels are lowest in torpor, but increase during IBA (Figure 4). NMDA receptor transcripts (*GRIN2B*, *GRIN3A*) are upregulated during October, torpor, and IBA as well.

The fact that there is evidence of both pre- and postsynaptic changes suggests global neurite retraction, and also provides more protection. This is because if one component of the synapse fails to exhibit appropriate plasticity, the other component would still provide protective dissociation. During the low body temperatures of torpor, both transcription [30] and translation [31] are suppressed to low levels, but as the animal begins an arousal from torpor and body temperature begins to rise, rates of transcription and translation presumably increase. While there was no net protein loss observed in the cerebral cortex synapses from torpor to IBA [7], the observed presence of proteolysis suggests that protein synthesis is occurring to replace the lost proteins, requiring transcripts that are either already present from the previous IBA or transcribed upon arousal. An analysis of synaptic cytoskeletal gene and protein expression over the course of an arousal from torpor could provide a valuable illustration of mechanisms of rapid plasticity and remodeling, specifically how synapses reform and whether maturation of one side of the synapse precedes the other.

#### Extracellular Matrix Modification

The cerebral cortex transcriptome also reveals some modulation outside of the cells that could contribute to synaptic plasticity and morphogenesis, possibly creating a scaffold for motile neurites, stabilizing the synapse, or serving as a physical barrier of signal transduction. The ECM of the adult brain in general is distinct from other areas of the body in that it lacks many structural, fibrous proteins [32]. However, in the hibernating ground squirrel cerebral cortex, there are elevated transcript levels of collagens and laminins during hibernation, particularly in torpor (Figure 5A), more characteristic of the plasticity seen during development, where these structural components are involved in axon guidance and neurite extension [32]. Additionally, other ECM components are also elevated, including *HAPLN2*, integrins, and notably agrin, which is important for synapse organization [33]. Because of the plasticity seen in the hibernator brain, these ECM components could be playing a role similar to their role in development. ECM components are also known to be involved in short-term synaptic plasticity and receptor organization [34]. In addition to transcripts of ECM structural components, elements of ECM breakdown are also elevated during hibernation (torpor and IBA), including members of the matrix metalloproteinase family (*ADAM15*, *ADAMTS9*), which could play a role in the plasticity and turnover of the brain ECM components as animals cycle between torpor and IBA [35]. The restrictive nature of the adult ECM in widespread reorganization and regeneration represents a major hurdle clinically [32], so the hibernator brain could contribute greatly to understanding mechanisms of ECM plasticity for therapeutic implementation.

#### Lack of hypothalamic plasticity

Transcriptional evidence of synaptic plasticity is not seen in hypothalamus during hibernation, or at least not to the same extreme as in the cerebral cortex. *MAP1B* is elevated during October, torpor, and IBA in the hypothalamus, but to a lesser degree than in the cerebral cortex (Table S1). Extracellular matrix proteoglycan transcripts versican (*VCAN*) and aggrecan (*ACAN*) are also elevated during hibernation in hypothalamus, but not cerebral cortex. Interestingly, *ACAN* is almost exclusively found in plasticity-restricting perineuronal nets in the brain [36], indicating that these extracellular matrix structures could be preventing plasticity in the hypothalamus during hibernation. *ACAN* is also elevated in pre-hibernation, indicating that it could be involved in preparing the hypothalamus for hibernation. Many plasticity-associated components elevated during hibernation in the cerebral cortex actually show the opposite expression pattern in hypothalamus, with higher expression levels during the pre- and post-hibernation collection points (Table S1). Thus, it is possible that restructuring of the neurons and ECM in the hypothalamus occurs prior to the onset of hibernation, after completion of hibernation as a recovery mechanism, or both.

### Mechanisms of Repair in the Hypothalamus

While the hypothalamus transcriptome displayed little indication of remodeling or plasticity during hibernation, it did reveal an array of defense mechanisms centered on survival, repair, and regulation of protein turnover (Figure 6). This suggests that the hypothalamus might sustain damage during hibernation, possibly due to the fact that it continues to function throughout torpor and IBA [3]. The data presented here suggest that protective strategies are in place in the hypothalamus throughout hibernation. Additionally, a previous study reported that no neuropathological abnormalities were noted in any brain area during torpor or IBA [2], again suggesting that protective mechanisms are in place.

Transcript levels for a variety of survival signals and DNA repair components are elevated during hibernation in the hypothalamus. *MEF2D*, *STAT3*, and B-cell CLL/lymphoma 6 (*BCL6*) have all been shown to promote survival of neurons [37], [38], [39]. *MEF2D* and *STAT3* show the highest expression during IBA, while *BCL6* is elevated in October, torpor, and IBA (Figure 6A). *RAD50* and nibrin (*NBN*) are part of a repair complex that senses double strand DNA breaks and recruits DNA repair coordinator *ATM* [40]. *RAD50* is elevated during torpor, while *NBN* and *ATM* are elevated during both torpor and IBA (Figure 6A). *ATM* is upregulated during the hibernation collection points in the cerebral cortex as well, indicating that this could be a shared mechanism of protection. *RAD50* is one of the most abundant transcripts in the cerebral cortex (Figure 3) and is also differentially expressed, but shows the opposite expression pattern: elevated during the pre- and post-hibernation collection points (Table S1). It is possible that the DNA damage response is not as crucial in the cerebral cortex due to the extensive plasticity that occurs.

In addition to repair mechanisms, the hypothalamus also appears to implement ubiquitin-dependent protein catabolism (Figure 6B). Ubiquitination is a post-translational modification that marks proteins for degradation and recycling at the proteasome. E3 ubiquitin ligases *UBR1* and *UBR5* are upregulated during hibernation (torpor and IBA), along with deubiquinating *USP12*, *USP16*, and *USP32* (Figure 6B). Ubiquitination can also serve other functions, for instance, *UBR5* has been implemented in DNA damage response [41]. The ubiquitin-proteasome system has also been shown to play a role in protection by degrading cell death promoting proteins [42]. Transcripts involved in the process of translation to replace degraded proteins are elevated during hibernation as well, including *EIF2A*, elevated in torpor, and *RBM3*, which has the lowest expression in April (Figure 6B).

### Transcripts Contributing to Hibernation Physiology

#### Hibernation seasonal induction

*VGF* mRNA is upregulated in the arcuate nucleus of the hypothalamus in response to a switch from long to short photoperiod in hamsters [43], suggesting that expression of this gene could play a role in relaying seasonal information. This transcript is elevated in the pre-hibernation October collection point in the hypothalamic transcriptome in comparison to all other collection points (Figure 7A), and therefore could be providing pertinent hibernation timing information to the ground squirrels. *VGF* is also upregulated during October in the cerebral cortex, but it is not as highly expressed as in hypothalamus (Figure 7B). *VGF* has also been implicated in body weight regulation, food intake, and energy balance [44], suggesting it could be extremely important for seasonal induction of hibernation. Similarly, *TRH* also peaks in October in the hypothalamus transcriptome. *TRH* is known to be involved in cold stress and inhibition of feeding [45], and could also be playing an important role in hibernation induction.

#### Torpor maintenance and arousal timing

Adenosine signaling appears to be very important for seasonal torpor induction. Administration of an adenosine A1 receptor antagonist reversed spontaneous entrance into torpor, while the agonist induced torpor in arctic ground squirrels during the hibernation season [46]. While we did not see differential expression of the adenosine receptors, adenosine kinase (*ADK*) exhibited elevated expression during hibernation (torpor and IBA) and lowest expression during the pre-hibernation October collection point in the hypothalamus (Figure 7A). *ADK* regulates extracellular adenosine levels by converting excess adenosine to AMP. This mechanism could potentially contribute to the timing of IBAs by removing the source of adenosine fueling torpor maintenance or by increasing levels of AMP, which was previously shown to induced torpor in mice [47].

Circadian clock gene rhythms have been proposed to underlie torpor and IBA timing, but previous work indicates that these rhythms are suppressed during torpor [48]. However, the suprachiasmatic nucleus (SCN) of the hypothalamus, which contains the master circadian clock, remains active throughout hibernation. The SCN showed increasing *c-fos* expression over torpor, with the highest expression in late torpor and early arousal [3]. These animals were kept in constant darkness, so this neuronal activity was not the result of environmental influence. Additionally, lesioning the SCN in ground squirrels altered seasonal hibernation timing and torpor bout timing [11], suggesting that this brain region could be very important in the timing of arousals and also in seasonal induction of hibernation. Examination of clock gene expression in the hypothalamic transcriptome reveals that two genes are differentially expressed: *ARNTL* and *BHLHE40* (Figure 7A). *ARNTL*, also known as *BMAL1*, has low expression in April compared to all other collection points. This gene is necessary for rhythmicity of the circadian clock [49]. *BHLHE40* is elevated during torpor compared to all other collection points, suggesting that it could play a role in torpor bout timing.

#### Energy metabolism

Previous work indicates that the brain preferentially utilizes ketones as fuel during hibernation, even in the presence of glucose [22]. The transcript for the ketone transporter *SLC16A1* (also known as MCT1) displays the lowest expression in April in both cerebral cortex and hypothalamus (Figure 7), confirming previous findings and providing evidence of molecular preparation of the fuel switch prior to hibernation onset. Genes involved in fatty acid beta-oxidation (*ACAD10*, *ACADVL*, and *ACACB*) are elevated in the cerebral cortex in hibernation, with the highest levels in torpor (Figure 7B), further validating this preferential fuel source. Glucose conservation through downregulation of glycolysis in the cerebral cortex during hibernation is supported by low expression of *ALDOC* and high expression of *PFKFB2* in both torpor and IBA (Figure 7B). Reduced glycolysis during hibernation in the brain was previously reported [22]. *ALDOC* is an enzyme in the glycolysis pathway, while *PFKFB2* can both synthesize and degrade fructose-2,6-bisphosphate, which regulates both glycolysis and gluconeogenesis. Low *ALDOC* protein expression was previously reported during hibernation in the brainstem [50]. However, there is some evidence of glucose use in the cerebral cortex during hibernation, with the elevation of *GAA* (Figure 7B), which is responsible for the degradation of glycogen to glucose.

In contrast to the transcriptional evidence of ketone utilization and glucose conservation seen in the cerebral cortex, the continuously active hypothalamus does appear to utilize glucose as a fuel source during hibernation, at least part of the time. *LDHA* mRNA is elevated during hibernation in the hypothalamus (Figure 7A). LDHA is the last enzyme in anaerobic glycolysis, suggesting that the hypothalamus is using glucose as an energy source during the low oxygen periods of torpor. *LDHA* elevation in hibernation was previously reported in brown adipose tissue [51], but not in any brain region. This mechanism of energy production could be unique to tissues that are active during torpor and arousal. Evidence of beta oxidation in the hypothalamus during hibernation is also present, with expression of *ACSS2*, which shows the lowest expression in April compared to all other collection points (Figure 7A).

One of the most abundant genes in hypothalamus, *FABP4*, could also be playing an important role in energy metabolism. It is also differentially expressed, with the highest levels in October (Figure 4). *FABP4* is an adipocyte specific fatty acid binding protein, but inactivation of this gene with Cre recombinase in mice demonstrates that the central nervous system is also affected [52]. High levels of *FABP4* in the hypothalamus during pre-hibernation could be important for preparing the brain for the upcoming fuel switch accompanying hibernation. *FABP4* is also very abundant in the cerebral cortex, and thus could be important for hibernation globally in the brain.

#### Adaptations to cold

*RBM*3 enhances global protein synthesis at mildly hypothermic body temperatures [18]. It has also been shown to be necessary for the neuroprotective effects of therapeutic hypothermia [53]. This transcript displays the lowest expression in April in both cerebral cortex and hypothalamus (Figure 7). Rates of translation are very low during torpor [54], so this is most likely important for production of vital new proteins during arousal, when body temperature is increasing from the near-freezing torpor temperatures. Expression of this gene during hibernation has been previously reported in hypothalamus [55].

#### Satiety and feeding behavior

Ground squirrels consume large amounts of food in the spring after emergence from hibernation and throughout the summer to build fuel stores for hibernation. However, in the period of time preceding the hibernation season, their feeding declines and they do not eat any food during hibernation, even if it is offered artificially in the lab [9]. In general, our transcriptional data support the role of classic orexigenic (*AGRP*, *NPY*) and anorexigenic (*CARTPT*, *TRH*) genes in terms of hibernation feeding behavior (Figure 7A).

Our hypothalamic transcriptome analysis also revealed other transcripts that could be associated with feeding behavior, satiety, or obesity (Figure 7A). Bardet-Biedl syndrome 1 (*BBS1*) and proprotein convertase subtilisin/kexin type 1 inhibitor (*PCSK1N*) show the same expression pattern as *HCRT*, high outside of hibernation and low in the hibernation collection points. Mutations in *BBS1* are involved in the obesity-featuring Bardet-Biedl syndrome and this transcript has been implicated in fat storage in *C. elegans* [56]. Overexpression of *PCSK1N* in mice resulted in obesity and diabetes [57] and *PCSK1N*-derived peptides promote food intake [58].

### Pre-hibernation – Molecular Preparation for Hibernation

Ground squirrels physically prepare for the upcoming season by greatly increasing white adipose stores in the summer months [54]. Interestingly, the transcriptomes from both brain regions indicate that some genes elevated during hibernation are also elevated during the pre-hibernation October collection point (Table S1). In the cerebral cortex, this includes upregulation of transcripts needed in remodeling and plasticity, while in the hypothalamus, this includes transcripts involved in the DNA damage response and proteolysis, along with some shared transcripts like *SLC16A1* and *RBM3*. This indicates that preparation for hibernation in the brain occurs on the molecular level prior to hibernation onset, or in this case, prior to exposure to torpor-inducing conditions, such as removal of food, cold temperatures, and constant darkness. This also indicates that genes involved in the hibernation phenotype are not expressed as a reaction to hibernation conditions, but instead are most likely expressed due to an internal seasonal timing mechanism.

### Region-specific mRNA Abundance and Gene Expression Specificity

Comparison of overall mRNA diversity reveals that the cerebral cortex and hypothalamus are over 90% similar (Tables 1 and S1). Additionally, examination of the most abundant genes (Figure 3) indicates that the majority of the highly expressed genes are the same in both brain regions, and possibly globally throughout the brain. The most abundant transcripts presented here support a previous examination of transcript abundance in mouse parietal cortex and hypothalamus, where glyceraldehyde-3-phosphate dehydrogenase (*GAPDH*) was found in the top ten most abundant transcripts in both tissues [59]. Additionally, *CKB* was previously reported to be abundant in mouse parietal cortex and *SPARC* in mouse hypothalamus as well. *PTGDS* and *GNAS* were previously reported as abundant in mouse hypothalamus, but not parietal cortex, whereas we report both transcripts as abundant in the ground squirrel cerebral cortex sample, which includes parietal cortex. This could be because only the top ten most abundant transcripts in each region were reported previously, which included mitochondrially encoded transcripts that were excluded from our analysis.

Although the most abundant transcripts and overall mRNA diversity are very similar between hypothalamus and cerebral cortex, these regions do differ in gene expression specificity. In our analysis, the hypothalamus exhibits six times as many mRNAs that are region-specific compared to the cerebral cortex (Figure 2). Some of the hypothalamus-specific mRNAs reported here support a previous examination of gene expression specificity in specific regions within the hypothalamus in mouse [60]. For example, *HCRT* was so highly specific to the dorsomedial nucleus, that it is considered a marker of this region. Similarly, *CGA* is a marker for the median eminence. *OXT* and *AVP* were highly expressed in several hypothalamic nuclei, but in no other brain region. Kasukawa et al. [60] also examined gene expression specificity in many other regions throughout the brain, including sub-regions of the cerebral cortex. However, the mRNAs that show the highest specificity for cerebral cortex reported here do not match the region-specific markers reported earlier [60]. This is possibly because we examined global changes in cerebral cortex gene expression, rather than individual regions. Also, we only examined the transcriptomes of two brain regions, instead of regions throughout the brain, so our comparison did not take as many different parts of the brain into account. However, this does support the idea that the hypothalamus exhibits greater gene expression specificity than cerebral cortex.

### Conclusions

Analysis of the cerebral cortex and hypothalamus transcriptomes reveals that while these two brain regions are very similar in terms of mRNA diversity, they are regulated very differently during hibernation. Transcriptomic evidence indicates that the cerebral cortex undergoes plasticity and remodeling during torpor and IBA, which is not evident in the hypothalamus. Several plasticity-related transcripts elevated during hibernation in the cerebral cortex are upregulated before or after the hibernation season in the hypothalamus, suggesting that remodeling in this brain region occurs either as a preparatory or recovery mechanism. Evidence from the hypothalamic transcriptome suggests that this brain region implements a DNA damage response protective strategy, along with protein turnover through ubiquitination. Several DNA damage and proteolysis genes show the opposite pattern of expression in the cerebral cortex, with higher levels outside of hibernation. This could be because the cerebral cortex undergoes extensive plasticity and therefore does not need the upregulated defense responses during hibernation. Additionally, this analysis reveals that the brain prepares for hibernation on a molecular level prior to the onset of the season and provides some clues about how hibernation could be induced and maintained.

## Materials and Methods

### Animals

A total of 12 male and 12 female thirteen-lined ground squirrels (*Ictidomys tridecemlineatus*) were used in these experiments. All animals were housed individually in the AAALAC-accredited Animal Care Facility located in the University of Minnesota Duluth School of Medicine. The squirrels were kept at room temperature in a 12∶12 light/dark (LD) cycle and fed standard rodent chow and water *ad libitum* from April through October. During the hibernation season (November-March), the squirrels were moved into an environmental chamber and kept in constant darkness at 5–7°C with no food provided. All experimental procedures were approved by the University of Minnesota Institutional Animal Care and Use Committee (protocol #1103A97712).

### Experimental Collection Points

The collection points for these experiments were chosen to uncover the most meaningful comparisons in the ground squirrel brain across the hibernation season. Four collection points (Figure 1) were used: pre-hibernation (October active), Torpor, IBA, and post-hibernation (April active). Three males and three females were sacrificed at each collection point. Animal state at each collection point was verified by rectal body temperature and animal behavior (Torpor: 6–8°C/inactive; Active/IBA: 35–37°C/active). All animals were sacrificed between 10 am and 3 pm.

The October collection point provides a time when the animals are already physically prepared for hibernation, having doubled or even tripled their body weights in the preceding months [54]. This collection point represents an opportunity to examine how the brain prepares for the oncoming hibernation season. Ground squirrels can potentially have begun torpor bouts in October [61], and because body temperature was not continuously monitored during this time, it is possible that some or all of the animals had already undergone torpor bouts. However, all the animals used for this collection point were held on a 12∶12 LD cycle at 23°C, had access to food, and were active at the time of collection. Pre-hibernation animals were sacrificed in the first two weeks of October.

The torpor and IBA collection points provide the two extremes of the hibernation cycle. In torpor, the ground squirrels exhibit near freezing body temperatures, low heart rates, and low oxygen consumption, while all of these physiological parameters return to normothermic levels at IBA. The transition from torpor to IBA takes an average of 2.8 hours in the thirteen-lined ground squirrel [23], meaning that the molecular mechanisms orchestrating the transition to normothermia and activating the underlying neuroprotective strategies are rapidly implemented and/or sustained during this process. In an effort to keep transitions between torpor and IBA as natural as possible, the animals were not handled and body temperature was not rectally assessed prior to sacrifice, as this could affect arousal parameters or trigger a premature and/or unnatural arousal. Therefore, the exact timing of sacrifice within the torpor bout and IBA is not known. However, all animals were monitored daily using the sawdust method [62], and the animals used for the torpor collection point were at least three days into a torpor bout and had shown no signs of arousal. Torpid state was verified at sacrifice by rectal body temperature (6–8°C). All animals used for the IBA collection point aroused naturally and spontaneously, were observed as awake and active (eyes open and exhibiting coordinated movements), and were torpid the previous day. All IBA collection point animals had a normothermic body temperature (35–37°C), verified by rectal measurement at the time of sacrifice. The hibernation collection point animals (Torpor and IBA) were sacrificed during January and February when average torpor bout length is longest.

Finally, the post-hibernation collection point in April provides an opportunity to examine the period of recovery after the completion of an entire hibernation season, and also represents the point of time when the ground squirrels are farthest from entering the next hibernation season. Post-hibernation animals were sacrificed in the second and third weeks of April. These animals had been removed from the hibernation chambers at the end of March and were held in the 12∶12 LD cycle at 23°C with *ad libitum* access to food.

### Brain Dissection

All animals were fully anesthetized with Isoflurane and then sacrificed by decapitation. The brain was removed from the skull and the meninges and blood vessels surrounding the brain were removed. The hypothalamus, located on the ventral side of the brain caudal to the optic chiasm, was removed first, avoiding the pituitary gland. The cerebral cortex was then removed using previous dissection methods [63], [64] and a rodent brain atlas as guides [65]. First, the olfactory bulbs, brainstem, and cerebellum were removed. The two cortical hemispheres were then separated by cutting through the corpus callosum. The hippocampus is layered directly under the cerebral cortex, separated by the lateral ventricle, so this was carefully extracted, along with the underlying thalamus. In an effort to remove all of the hippocampus, parts of the piriform cortex and portions of the most caudal regions of the entorhinal, ectorhinal, perirhinal, retrosplenial, and visual cortex could have been removed as well. The basal ganglia were then removed from below the frontal cortex. Finally, the white matter layer directly under the cerebral cortex, including the external capsule and cingulum, was removed, but it is likely that small portions of these structures were included in the dissected samples. All dissections were performed on ice and the dissected brain regions were rapidly frozen in liquid nitrogen. The two cortical hemispheres were frozen and stored separately. The time from decapitation to sample freezing was less than 10 minutes. Tissues were stored at −80°C until RNA purification.

### RNA Preparation

RNA was purified from hypothalamus and cerebral cortex samples using a Qiagen RNeasy Mini Kit and any remaining genomic DNA was removed with a DNase kit (Ambion). In an effort to determine global transcriptomic changes in the cerebral cortex rather than focusing on one specific region, one entire hemisphere from each animal was used for RNA purification. The other hemisphere was saved for future experiments. The entire bilateral hypothalamus sample from each animal was used for RNA purification. RNA samples from individual animals were quantified on a nanodrop and run on a gel to ensure good quality. RNA from one male and one female from each collection point were combined together into a single sample, so that each of the four collection points had 3 pooled samples. This pooling was done to control for any influence of sex. Because hibernation is a survival adaptation used by both sexes, we chose to eliminate sex as a possible variable in our data. Samples were then sent to the University of Minnesota Biomedical Genomics Center (St Paul, MN) for Illumina HiSeq 2000 sequencing.

### cDNA Synthesis and Illumina HiSeq 2000 Procedure

#### Sample Quality Assessment

Total RNA isolates were quantified using a fluorimetric RiboGreen assay. Total RNA integrity was assessed using capillary electrophoresis, generating an RNA Integrity Number (RIN). All of the 24 samples (12 from hypothalamus and 12 from cerebral cortex) were verified as high quality (>1 microgram, RIN = 8+), and thus were converted to Illumina sequencing libraries.

#### Library Creation

RNA samples were converted to sequencing libraries using Illumina’s Truseq RNA Sample Preparation Kit (RS-122-2001). In summary, 1 microgram of total RNA was enriched for mRNA using oligo-dT coated magnetic beads, fragmented, and reverse transcribed into cDNA. The cDNA was fragmented into smaller pieces, blunt-ended, and ligated to indexed (barcoded) adaptors and amplified using 15 cycles of PCR. Final library size distribution was validated using capillary electrophoresis and quantified using PicoGreen fluorimetry and qPCR. Libraries were successfully sequenced for all samples.

#### Cluster generation and sequencing

Truseq libraries were hybridized to a paired-end flow cell and individual fragments were clonally amplified by bridge amplification on the Illumina cBot. Libraries were clustered at a concentration of 12 pM. The flow cell was then loaded on the HiSeq 2000 and sequenced using Illumina’s Sequencing by Synthesis (SBS) chemistry. Upon completion of a read, a 7 base pair index read was performed for sample identification. Samples were run for 100 cycles with 10 million single reads per sample.

#### Primary analysis and de-multiplexing

Base call (.bcl) files for each cycle of sequencing were generated by Illumina Real Time Analysis software. The base call files and run folders were then exported to servers maintained at the Minnesota Supercomputing Institute (Minneapolis, MN). Primary analysis and de-multiplexing were performed using Illumina’s CASAVA software 1.8.2, resulting in de-multiplexed FASTQ files.

### Bioinformatics and Data Analysis

Raw reads generated (over 10 million per sample) were mapped to a set of *I. tridecemlineatus* contigs assembled in the open-source program Trinity [66]. A contig is a set of overlapping reads combined together, which can then be identified as a consensus region of RNA. The contigs were constructed using this data and previous *I. tridecemlineatus* RNAseq experimental data [67]. Trinity was used to predict coding domain subsequences within the contigs in order to specifically select for protein-coding transcripts. Any contig containing a predicted coding domain was selected and trimmed to include only that domain plus up to 100 bases on both ends. Additionally, mitochondrially-encoded reads were screened out using the thirteen-lined ground squirrel mitochondrial sequence assembled previously [67] and NCBI megablast. The selected contigs were then identified by comparison to the human RefSeq nucleotide database (NCBI) using Blastn (E-value of 10^−5^). This database was used instead of the recently sequenced ground squirrel genome [68], because the *I. tridecemlineatus* genome only includes predicted coding domains taken from comparison to the human sequence. Thus, comparing to the human RefSeq nucleotide database directly eliminates extra steps. However, the ground squirrel genome was used to supplement this identification for any contigs that needed species-specific sequence verification. Raw reads from each experimental sample were identified using these contigs, and then quantified into counts for each gene. Gene names used for identification are the official Human Genome Organisation (HUGO) Gene Nomenclature Committee (HGNC) designations.

Resulting counts were upper quartile normalized and then fitted to a negative binomial distribution using DESeq v1.6.1 [69]. All genes included in the initial analysis of each brain region had at least 10 counts total. Maximum fold change for each gene was calculated as the collection point with the highest average counts divided by the collection point with the lowest average counts. Fold changes calculated for the heatmaps and supporting information (Figures 5, 6, 7, Table S1) all use the April collection point as a baseline. The mean of each collection point was divided into the April mean to calculate the fold change. April expression is equal to 1. For purposes of clarity, fold changes that were less than one (expression is lower in April) were converted for presentation in the figures by taking the negative inverse. Each mRNA was also given a specificity value for each brain region, calculated as the percentage of counts in that region divided by the total number of counts in all other transcriptomic samples. The specificity value takes into account expression values from hypothalamus and cerebral cortex, along with white adipose tissue, brown adipose tissue, skeletal muscle, and heart, which were obtained from additional transcriptomic experiments [67] and unpublished data. Finally, all counts across all collection points were quantified for each mRNA to determine overall abundance in each brain region.

### Statistical Testing

Differential expression in each brain region was determined using an analysis of deviance in DESeq (command: nbinomGLMTest) to generate a test statistic (P-value) using the methods described by Anders and Huber [69], [70]. Each collection point consisted of 3 pooled samples for each brain region. The computed P-values were independently filtered [71] by restricting to those with at least a 50% change between any two collection points and at least one collection point with a mean of 100 or more reads. The Benjamini-Hochberg method was then used to correct for multiple comparisons [72], providing a P-value cutoff for significance which controlled the FDR at 0.05. The P-value cutoffs obtained from this procedure were 0.028 for the cerebral cortex and 0.039 for the hypothalamus. For each brain region, any transcript with a P-value less than the respective cutoff value was considered differentially expressed (FDR<0.05). All differentially expressed genes for each brain region are listed in Table S1, along with their means, standard errors, fold changes (in relation to the April collection point), and P-values. On these differentially expressed genes (cerebral cortex: n = 1085; hypothalamus: n = 1063), post hoc pair-wise comparisons were performed using the same function in DESeq (command: nbinomGLMTest), but with different input data. For this pair-wise analysis, the P-values were independently filtered [71] to restrict to a 50% change between two specific collection points, rather than any two. The Benjamini-Hochberg method was again used to control the false discovery rate (FDR) to 0.05 to correct for multiple comparisons [72]. P-values resulting from these pair-wise comparisons are listed in Table S2. Despite meeting significance criteria for differential expression in the previous analysis, not all mRNAs showed significant differences in the more conservative pair-wise comparisons, therefore Table S2 only includes mRNAs that resulted in at least one significant pair-wise difference between collection points. The P-value threshold values for significance for each pair-wise comparison in each brain region are listed in Table S3. P-values less than the threshold for each comparison were considered significant.

### Functional Analysis of Expression Patterns

The differentially expressed transcripts from each brain region (cerebral cortex: n = 1085; hypothalamus: n = 1063) were analyzed using the functional annotation tools of DAVID [17] and accompanying literature searches. Differentially expressed transcripts were sorted based on the collection point with the highest expression, and also independently sorted by highest expression values in hibernation (torpor and IBA) versus outside of hibernation (April and October), and these lists were entered into DAVID for analysis. DAVID analysis provided both functional annotation of each individual gene (Functional Annotation Table) and of each entered list of genes (Functional Annotation Clustering), which gave functional information using gene ontology [73], the Kyoto encyclopedia of genes and genomes (KEGG) pathway database [74], and information about human genetic disorders and syndromes from the Online Mendelian Inheritance in Man (OMIM) database [75]. The Functional Annotation Clustering tool provided an overall picture of the overrepresented and enriched functions in each brain region during hibernation and outside of hibernation, along with functions associated with individual collection points. Additionally, information on specific individual genes was obtained using the Functional Annotation Table tool and the scientific literature.

## Supporting Information

Table S1 Differentially expressed genes. Table provides the mean (n = 3) and standard error for each of the four collection points of all genes that are differentially expressed in cerebral cortex (sheet A) and hypothalamus (sheet B). The p-value generated from the DESeq analysis of deviance is also provided for each gene. A gene was considered differentially expressed and included in this table if at least one collection point had at least 100 total counts, there was at least a 50% change between the collection points with the highest and lowest counts, and the FDR was less than 0.05. The p-value cutoffs for significance (FDR<0.05) obtained from the Benjamini-Hochberg method were 0.028 for cerebral cortex and 0.039 for hypothalamus. Fold changes were calculated relative to the April time point. Gene names listed are the official HGNC designations. APR: April collection point; C: cerebral cortex; FC: fold change; FDR: false discovery rate; H: hypothalamus; IBA: Interbout arousal collection point; OCT: October collection point; TOR: Torpor collection point; SE: standard error.
(XLS)

Table S2 Pairwise comparisons of all differentially expressed genes. This table provides the p-values for each pairwise comparison of the differentially expressed genes listed in Table S1 that show at least one significant difference between collection points for cerebral cortex (sheet A) and hypothalamus (sheet B). The p-value cutoffs for significance (FDR<0.05) for all pairwise comparisons are provided in Table S3. APR: April collection point; C: cerebral cortex; FDR: false discovery rate; H: hypothalamus; IBA: Interbout arousal collection point; OCT: October collection point; TOR: Torpor collection point.
(XLS)

Table S3 P-value cutoffs for significance for pairwise comparisons. This table provides P-value cutoffs for significance (FDR<0.05) for all pairwise comparisons listed in Table S2 for cerebral cortex (sheet A) and hypothalamus (sheet B). These cutoff values were generated using the Benjamini-Hochberg method of correction for multiple comparisons. APR: April collection point, FDR: false discovery rate, IBA: Interbout arousal collection point, OCT: October collection point, TOR: Torpor collection point.
(XLS)
